# Is Cannabis Effective in the Treatment of Chronic Back Pain?

**DOI:** 10.7759/cureus.43220

**Published:** 2023-08-09

**Authors:** Josiah Damisa, Alexandra Petohazi, Hassan Jalil, Michelle Richardson

**Affiliations:** 1 Orthopedic Surgery, Royal Preston Hospital, Preston, GBR; 2 Trauma and Orthopedics, Royal Preston Hospital, Preston, GBR; 3 Orthopedic Surgery, University Hospitals of North Midlands NHS Trust, Stoke-on-Trent, GBR

**Keywords:** back pain, nhs-approved medications, chronic lower back pain, cannabis (marijuana), prescribed cannabis

## Abstract

Cannabis is commonly recognized as a recreational substance. It has been explored for its potential therapeutic applications in addressing various conditions, such as depression, anxiety, sleep disorders, neurological disorders, and chronic low back pain, which affect a significant portion of the population. In the United Kingdom, cannabis has been recognized and licensed for medical use since November 2018, with about 12 National Health Service prescriptions in circulation largely due to patient pressure, with support from media campaigns for its use when there was growing evidence of its use in intractable epilepsy. Cannabis is beginning to gain traction as an alternative or even a complementary drug to opiates with some pre-clinical studies showing opiate-sparing effects. Despite references to its therapeutic use, cannabis as a therapeutic drug has been controversial due to the negative perception of its use as a recreational drug. As a result, there have been challenges in changing the perception of healthcare authorities and clinicians on the use of cannabis as a therapeutic tool for pain relief. The stigma associated with cannabis could be responsible for the paucity of randomized controlled trials on the efficacy of medical cannabis, further decreasing the credibility of the few trials conducted.

## Introduction and background

Cannabis is commonly known as a recreational drug with an estimated 160 million users worldwide for recreational purposes. It has also been proffered as medical therapy in the treatment of varied ailments, including depression, anxiety, sleep disorders, neurological disorders, and many health disorders [1].

The use of cannabis as a therapeutic treatment in pain control has been well documented in the literature. Chinese and Indian texts in ∼2,000 BC referred to its use as pain relief, and, later, Greek references by Galen mentioned cannabis as a medicinal source of pain treatment. The modern use of cannabis as pain relief has been denoted in its use by Queen Victoria in dysmenorrhea [2,3].

Chronic low back pain affects about 25% of the population, and the pathophysiology causing it can be varied, ranging from neuropathic, inflammatory, or nociceptive [4]. Chronic back pain is expected to become more prevalent as populations age longer and the risk of age-related orthopedic diseases increases. Hence, the need for alternative forms of therapy as symptomatic control, especially considering opiate dependency and addiction. Evidence of this is seen in the willingness of 30 countries to legalize cannabis for medicinal purposes [5].

Since November 2018, cannabis has been recognized and licensed for medical use with about 12 National Health Service prescriptions in circulation largely due to patient pressure, with support from media campaigns for its use in view of growing evidence of its use in intractable epilepsy. Currently, Epidyolex and Sativex are the only two cannabis-based medicines recommended by the National Institute for Health and Care Excellence for medical conditions, including spasticity in multiple sclerosis, chemotherapy-induced nausea and vomiting, and intractable epilepsy. However, there is still no recommended form of medical cannabis for chronic pain in the United Kingdom [1,6].

Research into cannabis as a medical therapy for pain secondary to rheumatoid arthritis has shown some promising results with a marked decrease in pain when compared to a placebo [7]. Despite these references to its therapeutic use, cannabis as a therapeutic drug has been controversial due to the negative perception of its use as a recreational drug along with its association with anxiety, depression, and psychotic illness, which has inadvertently slowed the research into its medicinal properties [8,9]. Therefore, there is still limited knowledge of the therapeutic dosage, frequency, preferred route of administration, and its efficacy as an alternative therapeutic drug. In this review, we attempt to identify and summarize known information about the pharmacological advantages over other types of analgesia, the efficacy of cannabis as pain relief, and gaps that still require further research.

## Review

Methodology

**Figure 1:**
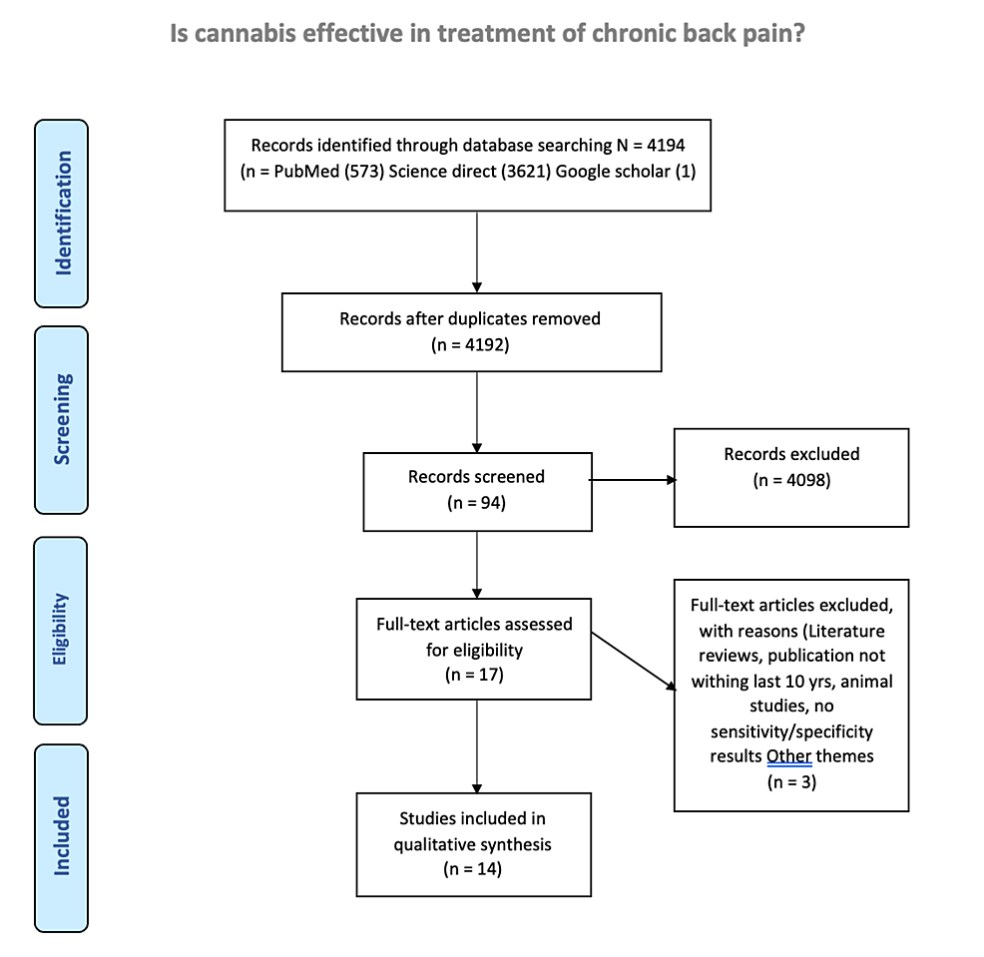
Study methodology.

A standard search of PubMed, Google, and Web of Science using listed keywords including cannabis, marijuana, cannabis and pain, cannabinoid and chronic pain, cannabis and low back pain was performed. To evaluate the efficacy of medical cannabis in chronic pain, any study of medical cannabis for other diseases not related to pain was excluded. Literature reviews, publications older than 10 years, and animal studies were excluded. Only peer-reviewed journal articles were included in this review. Studies were analyzed based on the indication for pain, the cannabis treatment modality used, and the study outcomes (Figure 1).

Pharmacology

While the primary mode of action of opioids is to inhibit pain by downstream mu-opiod receptor agonism, the role of cannabis in the modulation of pain sensation is currently understood through the prism of the endocannabinoid system [10]. Endocannabinoids are arachinoid acid derivatives which are produced by the neural and non-neural cells in response to stress such as tissue injury. Endogenous cannabinoids such as anandamide and 2-arachidonoyl-sn-glycerol activate the G protein-coupled cannabinoid (CB) receptors and subsequently suppress the sensitization and inflammation of these receptors [11].

The differing pathways of the mechanism of opioids and cannabis suggest a possible role for co-administration to induce analgesic effects. Nielson et al. in a meta-analysis of 19 preclinical studies showed that the co-administration of cannabis with opiates allows for lower doses of opiates without loss of analgesic effect, a phenomenon recognized as the opioid-sparing effect. The study showed the amount of morphine required to achieve the median effective dose was about 3.6 times lower when morphine was administered with cannabinoids compared to when morphine was administered alone [12].

The two main CB receptors, namely, CB1 and CB2, are activated by endocannabinoid agonists and the effect of this is the direct inhibition of neurotransmitters, including acetylcholine, dopamine, and glutamate, while also indirectly inhibiting gamma-aminobutyric acid, opioid serotonin, and N-methyl-D-aspartate receptors [13]. Ibrahim et al. showed that CB2 receptors in rats stimulate opioid precursor genes or stimulate the release of endogenous opioids, further enhancing anti-nociceptive properties [14]. This shows that the analgesic property of cannabis may also be derived by cannabis also stimulating the opiate pathway.

Efficacy of cannabis

Medicinal cannabis is available in several preparations and is administered through several routes. There appears to be only a small number of studies on the effects of cannabis on orthopedic issues such as arthritis, back pain, and post-trauma pain. Of the studies conducted so far, several systematic reviews have commented on the poor quality of methodology with studies having short follow-ups, small sample sizes, and assessing several forms of pain at same time [15].

In a systematic review of 24 research-controlled trials on inhaled cannabis, involving a total of 1,334 individuals, Aviram et al showed that while the majority of the studies showed inhaled cannabis results in improved neuropathic pain symptoms when compared to placebo, only two of the studies resulted in clinically significant results [16].

A systematic review of four randomized control studies by Madden et al. showed that oral nabilone showed no significant improvement in the relief of pain symptoms in orthopedic patients, and when compared to alternative analgesics, such as dihydrocodeine and ketoprofen, not only did Nabilone fare worse in the relief of pain symptoms but its side effects were worse than its comparatives. However, nabiximol oral spray when compared to placebo was shown to offer greater pain relief on the McGill and Numerical Rating Scale pain score. Generally speaking, Madden et al. showed that medicinal cannabis was more effective at higher doses, although there was no specificity as to what the optimal dose would be [17].

There is some anecdotal evidence that 5 g of cannabis offers some beneficial analgesic properties with the best ratio of maximal benefit to minimum adverse effect; however, there are still no standardized dosing standards for the prescription of cannabis with a high degree of variability among clinicians with prescription often tailored to patient’s response to pain control by cannabis [18].

It appears that most randomized controlled trials (RCTs) have focused on one form of medicinal cannabis with one single active agent when, as previously mentioned, medical cannabis is not made up of one medicinal agent but a combination of a group of agents. As suggested by Namdar et al., RCTs should focus more on medical cannabis with a combination of active agents, including tetrahydrocannabinol (THC) and cannabinoids, which would result in statistically significant positive results [19].

Another moot point, however, is the dependency of the scientific community on RCTs to determine the efficacy of medical cannabis. This apparent lack of confidence in cannabis efficacy is in contrast to the trust placed in the approval of about 50 medications by the Food and Drug Administration and the European Medicines Agency without RCTs between the periods of 1994 and 2008, but mainly based on the evidence from patient reports which place more emphasis on the well-being of the patient. Databases such as Project Twenty21 by Drug Science aim to create structured evidence for the efficacy of medical cannabis through patient-led reports. Patient-reported outcomes are now required for clinical trials funded by the National Institutes of Health [20,21].

Side effects of cannabis

The challenges with the use of cannabis in pharmacotherapy for pain control include the risk of one-tenth of users developing cannabis use disorder, which is characterized by psychosocial problems, increased drug-seeking behaviors, and physical symptoms such as craving, withdrawal, and tolerance [22,23]. Of note, acute intoxication and withdrawal symptoms of cannabis such as sleep problems and mood disturbance are similar to those observed in individuals with chronic pain [24].

Aviram et al. revealed that inhaled cannabis via pipe smoking resulted in the most adverse effects when compared with oral analogs such as dronabinol [16,25]. Acute cannabis use in the form of marijuana has been associated with a five times increase in road traffic accident-related deaths [26].

Chronic cannabis has also been associated with cognitive side effects which becomes an important factor for consideration, especially when prescribing medical cannabis for chronic pain.

In a systematic study of 23 RCTs on the adverse effects of medical cannabinoids, Wang et al. showed that the incidence of serious adverse effects, including respiratory and nervous system disorders, was not higher in patients who had medical cannabis versus controls; however, there was a higher incidence of non-serious adverse effects, including nervous system disorders, in cannabinoid users versus controls, especially when receiving oral THC. They deduced that the nervous system suffered the most adverse effects for both serious and non-serious adverse effects with psychiatric disorders as the next most common adverse effect [27].

Ethical issues

The most common route of administration of cannabis is currently via smoking due to its bioavailability and shorter half-life, with about 91% of individuals reporting this as the preferred route of administration in a study by Cranford et al. This raises several ethical conundrums for a physician prescribing it [28,29]. It also raises the problem of the effect of smoking on the respiratory system as well as its impacts on second-hand smoking problems with about 30-50% lost in side-stream. This is evident in the study by Herman et al. that showed second-hand smoking of cannabis results in low levels of THC and contributes to impaired psychomotor abilities and working memory [30]. This would be a dilemma for the physician who has sworn to *do no harm*.

There is still concern that due to the limited RCT data regarding the efficacy and safety of cannabis on specific clinic conditions rather than broad categories, such as chronic pain, it may impair the physician’s clinical decision and encourage prescription for vague indications with an element of chronic pain, rather than for an evidence-based condition, predisposing to overprescription of cannabis and potential abuse, especially when taking into account the psychotic side effects of cannabis [31].

Due to the historical association of cannabis as a recreational drug, there have been challenges in changing the perception of healthcare authorities and clinicians on using cannabis as a therapeutic tool for pain relief. This is evident as more than 70% of rheumatologists polled in Canada believed that cannabis has no role in conditions treated in rheumatology, with more than 75% indicating that they lacked the necessary knowledge about the efficacy of cannabinoids [32].

There have been fears that the medical use of cannabis will encourage more recreational use of this drug with a study in the United States showing that as acceptance of cannabis for medical use increases, there has been a corresponding increase among the youth for recreational purposes [33]. With the known psychotic effects of the recreational drug, there might be an increase in the incidence of the recreational form of cannabis as cannabis makes its way to unauthorized third-party groups [31].

Discussion

In the US states where cannabis has been legalized for medical use, there has been a corresponding decrease in the prescription of opioids and correspondingly lower opioids overdose mortality rates when compared with states that do not, with the reasoning that medical cannabis reduces the need for opioids in pain relief [34,35].

Cannabis is beginning to gain traction as an alternative or even a complementary drug to opiates with some preclinical studies showing opiate-sparing effects. However, it has to be stressed that these were mainly preclinical studies and results may not necessarily be the same in clinical studies. However, these results are promising and indicate that further clinical studies are required to determine if these effects are translated to humans.

Despite the early anecdotal assertions of the positive impact of cannabis on pain relief by several case reports, there is a paucity of studies, especially RCT, comparing the efficacy of cannabis and other active comparative analgesics. Instead, the efficacy has been evaluated by comparing it with a placebo; hence, very few deductions can be made about the use of cannabis as superior to or even as an alternative to other forms of analgesia currently available [31].

The stigma associated with cannabis can be responsible for the paucity of RCTs on the efficacy of medical cannabis, further decreasing the credibility of the few trials conducted.

In the majority of RCTs on the efficacy of cannabis, more emphasis has been placed on the medicinal effect and less on the side effects. Hence, more studies are needed that also evaluate the side effects alongside the medicinal properties of cannabis.

The ultimate aim will be to be able to use cannabis as an alternative and to eventually reduce the use of opioids; however, the evidence available is weak with respect to the efficacy in its application for chronic pain. However, there might be a role in individuals who have exhausted all other forms of standard treatment including opiates and for whom this will provide an alternative to ease the pain and to be considered as a form of a last-resort medical treatment.

## Conclusions

Cannabis is gaining attention as a potential alternative or complementary treatment to opioids, with preclinical studies suggesting opiate-sparing effects. However, it should be noted that these studies were primarily conducted on animals, and the applicability of these findings to human clinical studies remains uncertain. Thus, further research is needed to determine if these effects can be replicated in human subjects. Additionally, there is a scarcity of studies, particularly RCTs, comparing the efficacy of cannabis to other active analgesic treatments. Instead, most studies have focused on comparing cannabis to placebo, making it challenging to draw definitive conclusions about its superiority or viability as an alternative to existing forms of pain relief.

The limited number of RCTs exploring the medical efficacy of cannabis can be attributed to the stigma associated with its use. This stigma has hindered comprehensive research efforts, leading to a lack of robust evidence. Moreover, the emphasis in most RCTs has been on evaluating the medicinal properties of cannabis, while providing limited information on potential side effects. To address these gaps, further studies are needed that assess both the therapeutic benefits and potential adverse effects of cannabis. Although the ultimate goal is to utilize cannabis as an alternative to opioids, the current evidence regarding its effectiveness in managing chronic pain is inconclusive. However, in cases where individuals have exhausted standard treatment options, including opioids, cannabis may be considered as a last-resort medical option for pain relief.
